# The *MC1R *gene in the guppy (*Poecilia reticulata*): Genotypic and phenotypic polymorphisms

**DOI:** 10.1186/1756-0500-4-31

**Published:** 2011-02-04

**Authors:** Ayumi Tezuka, Hiroaki Yamamoto, Jun Yokoyama, Cock van Oosterhout, Masakado Kawata

**Affiliations:** 1 Department of Ecology and Evolutionary Biology, Graduate School of Science, Tohoku University, Sendai 980-8578, Japan; 2 Department of Developmental Biology and Neuroscience, Graduate School of Science, Tohoku University, Sendai 980-8578, Japan; 3 Department of Biology, Faculty of Science, Yamagata University, 1-4-12 Kojirakawa, Yamagata-City 990-8560, Japan; 4 Evolutionary Biology Group, Department of Biological Sciences, University of Hull, Hull, HU6 7RX, UK; 5 Faculty of Bioscience, Nagahama Institute of Bio-Science and Technology, 1266 Tamura-cho, Nagahama, Shiga 526-0829, Japan; 6 School of Environmental Sciences, University of East Anglia, Norwich NR4 7TJ, UK

## Abstract

**Background:**

The guppy (*Poecilia reticulata*) is an important model organism for studying sexual selection; male guppies have complex and conspicuous pigmentation, and female guppies exhibit preferences for males with specific color spots. Understanding the genetic basis underlying pigmentation variation in the guppy is important for exploring the factors causing the maintenance of color polymorphism in wild populations.

**Findings:**

We focused on the melanic black pigmentation of guppies, and examined genetic variations in the *melanocortin 1 receptor *(*MC1R*) gene because variation in this gene is known to contribute to polymorphism of melanin pigmentation in several animal species. The complete coding sequence of the guppy *MC1R *gene was determined, and two different *MC1R *alleles (963 and 969 bp) were found in wild populations. Ornamental strain guppies with a 963-bp *MC1R *tended to show less black pigmentation than those with a 969-bp *MC1R*, although the association between *MC1R *genotype and black pigmentation disappeared in the F_2 _offspring.

**Conclusions:**

The guppy *MC1R *gene showed variation in the five wild Trinidadian populations we examined, and these populations also differed in terms of allele frequencies. We identified a significant association between black pigmentation and *MC1R *genotype in fish obtained from aquarium shops. However, the results from F_2 _families suggest that there are other genes that modify the effects of the *MC1R *gene.

## Background

Pigmentation plays important roles in various aspects of several animal species, including camouflage, warning, thermoregulation, protection from ultraviolet radiation, and courtship display [1]. Therefore, individual variation in pigmentation can be a target of both natural and sexual selection, and thus mutations that change pigmentation influence adaptive evolution. Recently, identification of the genes responsible for pigmentation polymorphism has gained considerable attention in evolutionary biology [2].

The guppy (*Poecilia reticulata*) exhibits extreme pigmentation polymorphism in the secondary sexual traits of males, which is complex, conspicuous, and manifested as spots, speckles, and lines of various pigmented colors, including black, white, red-orange, yellow and green [3]. These fish also display iridescent structural color [3]. Female guppies show preferences for a specific type of male pigmentation, including orange and black, and various color spots [3]. Although directional selection such as specific female preference is expected to reduce variation in male traits [4], various color spots are maintained as polymorphic traits within populations [3]. Many studies have attempted to explain the maintenance of body color variation in the guppy and have suggested that maintenance of male pigmentation polymorphism is caused by negative frequency-dependent selection [5,6], selection maintaining multiple traits within populations [7], and/or gene flow with divergent selection [8]. Although several evolutionary mechanisms have been proposed, none has been conclusively demonstrated. One of the obstacles to elucidating the evolutionary mechanisms is the lack of information on the genetic basis of pigmentation, since the evolutionary responses to selection for the pigmentation depend largely on how the phenotypes are controlled by genes. For example, the selection force for coloration will be influenced according to whether the genes involved in melanogenesis show multiple pleiotropic effects [9].

Male guppies display many types of color pigment on their bodies. In this study, we particularly focused on a candidate gene that contributes to the polymorphism of black pigmentation. Black pigmentation in guppies plays two distinct roles, namely, in nuptial display and in camouflage. Females from populations with a higher proportion of orange coloration tend to have a stronger preference for orange and black [10]. In contrast, natural selection is predicted to operate against conspicuous black spots in habitats with visually hunting predatory fish (i.e., the so-called "high-predation sites") [11].

Black pigmentation is produced by melanin synthesis, which involves many genes. The coding sequence of one of these genes, *melanocortin 1 receptor *(*MC1R*), is reported to contain variations that are associated with melanin pigmentation polymorphism in natural populations in many animals [Reviewed in 2]. Among the pigmentation genes, *MC1R *plays a crucial role in controlling melanin synthesis [12]. In mammals and birds, high activity of *MC1R *leads to the synthesis of black eumelanin, whereas low activity leads to reddish pheomelanin, or an absence of melanin synthesis [13,14]. In contrast, fish melanophores contain only eumelanin and are unable to produce pheomelanin [15]. Thus, in guppies, like other fishes, lower activity of *MC1R *may lead to an absence of black pigmentation.

In this study, we focused on *MC1R *as a candidate gene that contributes to the polymorphism of black pigmentation in guppies. The purpose of this study was to determine the complete coding sequence of guppy *MC1R*, and to examine whether *MC1R *polymorphisms are present in wild guppy populations in contrasting habitats. We also examined whether sequence variation in *MC1R *affect black pigmentation by comparing *MC1R *genotypes and body color phenotypes.

## Materials and methods

### Determination of the complete coding sequences of guppy *MC1R*

We determined the coding sequences of *MC1R *using a wild male guppy that was caught from Okinawa Island in Japan and subsequently reared in our laboratory. Total RNA was isolated from the whole body of the guppy (except for the digestive organs) using TRIzol (Invitrogen, Paisley, UK) according to the manufacturer's protocol. cDNA was synthesized from the total RNA using a GeneRacer Kit (Invitrogen) according to the manufacturer's protocol.

In order to determine the 5' and 3' regions, 5' and 3' RACE was performed using the primers GeneRacer 5' Primer, GeneRacer 3' Primer, GeneRacer 5' Nested Primer, GeneRacer 5' Nested Primer (Invitrogen), PrMC1R-F1, PrMC1R-F2, PrMC1R-R1, andPrMC1R-R2 (Table 1). Amplification reactions followed PCR protocols in a volume of 50 μl, using 0.25 μl of the cDNA mixture, 5.0 μl 10× rTaq Buffer, 1 μM dNTP mixture, 0.5 μM MgCl_2_, 1 unit rTaq (Takara), and each primer. The amplification conditions were as follows: 2 min at 94°C; then 30 cycles of 30 s at 94°C, 30 s at 50°C, and 1.5 min at 72°C; ending with 15 min at 72°C. Semi-nested PCR was then performed with the PCR products, using the primers GeneRacer 5' Nested Primer, GeneRacer 3' Primer, PrMC1R-F1, PrMC1R-F2, PrMC1R-R1 and PrMC1R-R2 (Table 1). The semi-nested PCR products were separated by 1.5% SeaPlaque GTG Agarose (FMC BioProducts) gel electrophoresis and then gel purified.

**Table 1:** Primers used for cDNA synthesis, amplification, and sequencing

Primer	Sequence (5'-3')
PrMc1r-F1	CAAGAACAGSAATCTTCATTCRCCCATGTA
PrMc1r-F2	CCATCTTTTACGCRCTSCGGTACCACAG
PrMc1r-R1	CTGYGGTARGCGTADATGAGMGGGTC
PrMc1r-R2	AGRAAGAAVGGGCCCCAGCAGAG
PrMc1r-Fp1	TCTTCCCGTGGACTGATGAAACTTA
PrMc1r-Fp2	CAGATCCGCATCCGCAGGAGCTTTTC
PrMc1r-Rp1	CACGTCGACCTAATTTGCCTCAATTT
PrMc1r-Rp2	GGTCCGCGGCGATGGTGCACAGGAAGG

Semi-nested PCR products were sequenced using an ABI 3130 Genetic Analyzer (Applied Biosystems, Warrington, UK). The sequencing reactions were carried out using a BigDye Terminator v3.1 Cycle Sequencing Ready Reaction Kit (ABI) under the following conditions: 1 min at 96°C, then 45 cycles of 30 s at 96°C, 15 s at 50°C, and 4 min at 72°C. The primers used for the initial amplification were subsequently used for sequencing: GeneRacer 5' Nested Primer, GeneRacer 3' Primer, GeneRacer 3' Nested Primer, PrMC1R-F1, PrMC1R-F2, PrMC1R-R1, and PrMC1R-R2 (Table 1). Sequencing reaction products were purified and cleaned by ethanol precipitation. The sequences obtained were edited and aligned using Clustal X software [16].

### Determination of *MC1R *sequences in wild Trinidadian populations

**Figure 1:**
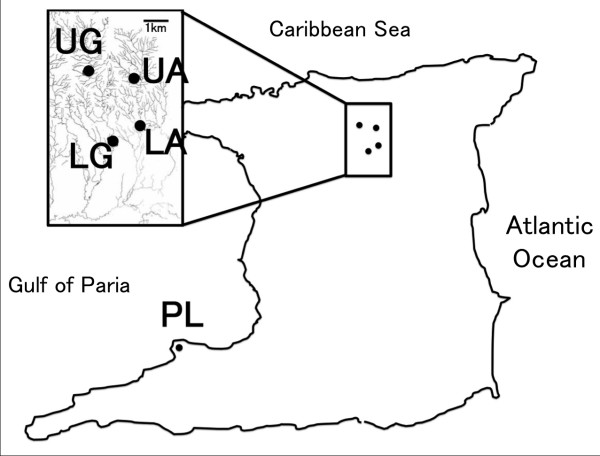
**Sampling locations in Trinidad**. UA, Upper Aripo River; LA, Lower Aripo River; UG, Upper Guanapo River; LG, Lower Guanapo River; PL, Pitch Lake

To examine whether *MC1R *polymorphism is present in wild guppy populations, we used tissue samples from 270 guppies that were collected from five wild populations on the island of Trinidad: Pitch Lake, Lower Aripo River, Upper Aripo River, Lower Guanapo River, and Upper Guanapo River (Figure 1). Because all samples were fixed with 99.5% ethanol before this study, we have no information regarding their natural pigmentation.

Total DNA isolation was performed according to the CTAB protocol [17]. On the basis of the cDNA sequences determined in this study, we designed primers from the *MC1R *untranslated regions: PrMC1R-Fp1 and PrMC1R-Rp1 (Table 1). Complete coding sequences of *MC1R *were amplified using these primers. Amplification reactions followed PCR protocols in a volume of 50 μl, using a 2.5 μl of the DNA mixture, 5.0 μl 10× rTaq Buffer, 1 μM dNTP mixture, 0.5 μM MgCl_2_, 1 unit rTaq (Takara), and each primer. The amplification conditions were as follows: 2 min at 94°C; then 30 cycles of 30 s at 94°C, 30 s at 50°C, and 1.5 min at 72°C; ending with 15 min at 72°C. The PCR products were purified and cleaned by PEG precipitation. Sequences were determined in both directions using an ABI 3130 Genetic Analyzer (Applied Biosystems). The sequencing reactions were carried out using a BigDye Terminator v3.1 Cycle Sequencing Ready Reaction Kit (ABI). Sequencing was performed under the same conditions as described previously. Sequencing was performed using the PCR primers and additional internal primers: PrMC1R-Fp2 and PrMC1R-Rp2 (Table 1). The sequences obtained were edited and aligned using Clustal X software [16].

### Association between *MC1R *genotypes and phenotypes

To confirm the association between phenotypes and *MC1R *genotypes, a total of 67 guppies were obtained from four aquarium shops. By choosing guppies that showed distinctly different pigmentation patterns, we ensured that fish from more than two different families were included in the sample obtained from each shop.

We evaluated black pigmentation using two traits: "body blackness," which is overall body blackness used in crypsis, and "black traits," which are conspicuous discrete black spots and lines on several parts of the body that are used as sexual signals. Although both male and female guppies display "body blackness," only the males express "black traits," which might be a secondary sexual trait in wild populations [3]. We evaluated the two traits independently because these traits might be controlled by different genes. Male-specific ornaments such as black spots and lines (i.e., black traits) are highly heritable between father and son, and thus certain sex-dependent genes (such as those on the Y chromosome) are assumed to affect black traits [3]. On the other hand, individuals of both sexes show whole-body blackness. Thus, we considered that the combinations of genes that affect black pigmentation in black traits and body blackness are different.

**Figure 2:**
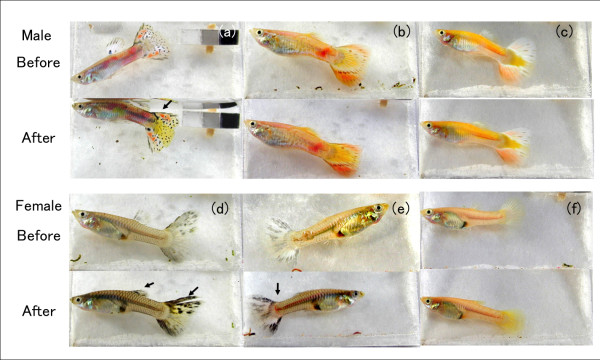
**Phenotypic variation among *MC1R *genotypes**. (a) *+/+ *male, (b) *+/Del *male, (c) *Del/Del *male, (d) *+/+ *female, (e) *+/Del *female, and (f) *Del/Del *female. For both males and females, the upper series of photographs ("Before") were taken before anesthesia, whereas the lower series ("After") were taken after anesthesia (see Methods). Arrows indicate black traits.

The chromatophores of fish are under neuroendocrine control, such that color and patterns can be changed almost instantaneously [18]. Therefore, the estimation of pigmentation was performed by taking photographs before and after anesthesia (Figure 2). We used phenoxyethanol at a 1:1500 dilution as an anesthetic. Anesthetized specimens showed melanin pigmentation that was similar to or darker than that in the un-anesthetized specimens (Figure 2). If an individual has melanin pigments and melanophores in its cells, anesthetic treatment cannot suppress melanin aggregation, and consequently, the body color becomes darker.

For body blackness, each guppy was categorized as either grayish or yellowish: "Grayish" guppies have black pigmentation whereas "Yellowish" guppies lack black pigmentation. The existence of black pigmentation is evident when the guppy is anesthetized. Thus, a guppy could be correctly categorized as yellowish when we failed to observe black pigmentation even under anesthetized conditions. If a guppy has melanin pigments, its body color becomes darker under anesthetized conditions. For black traits, each guppy was categorized as either black or none. "Black" guppies had more than one black spot and/or line. "None" guppies had no black spots or lines under both anesthetized and un-anesthetized conditions. Although the categorizations were performed by eye, we were confident that the guppies were correctly assigned since four persons independently scored the guppies and each of these individuals gave the same assessment.

After taking photographs, a part of the caudal fin was clipped from each fish for DNA isolation. All tissues were fixed with 99.5% ethanol and stored at -20°C until dissected. Genotyping was conducted using previously described protocols.

### Association between *MC1R *genotypes and phenotypes in F_1 _and F_2 _individuals

We found two different alleles of the *MC1R *gene (*Del *and +, see Results). F_1 _families were established by crossing individuals homozygous for the two different *MC1R *alleles: a *Del/Del *male obtained from an aquarium shop and a *+/+ *female derived from a wild population in Okinawa Island. We successfully obtained F_1 _offspring from two pairs. We raised the two families, and selected four pairs of F_1 _individuals (i.e., a total of eight F_1 _individuals; one pair in one family and three pairs in the other family). A total of 39 F_2 _individuals were obtained by two crosses of the F_1 _pairs. All F_2 _individuals were raised in groups of three to five fish per tank until 3 months of age. All the F_1 _and F_2 _fishes were maintained at 25°C with a constant photoperiod of 12 h light and 12 h dark, and were fed with Tetramin fish food. Genotyping and an estimation of phenotypes were performed using previously described protocols.

### Statistical analysis

Differences in frequencies of the different *MC1R *alleles among Trinidadian populations were analyzed using a G-test, and multiple comparisons of the allele frequencies of the populations were conducted using a simultaneous test procedure [19]. Deviation from Hardy-Weinberg equilibrium in the genotype frequencies was tested using a randomization test. Differences in frequencies of the different *MC1R *genotypes between Grayish and Yellowish body blackness and between Black and None black traits were tested using Fisher's exact test. The excess of heterozygous genotypes in F_1 _families was also tested using Fisher's exact test. The significant values were adjusted using sequential Bonferroni procedures. These statistical analyses were performed using the *R *statistical computing environment [20].

## Results

### DNA sequencing of guppy *MC1R *and *MC1R *polymorphism

**Figure 3:**
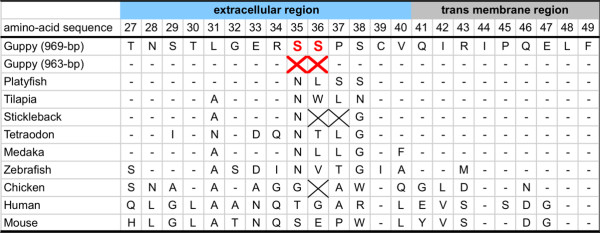
**Amino acid alignment in the extracellular region and the transmembrane region surrounding the deletion sites in MC1R**. The sites of the deletion mutation in guppy *MC1R *are highlighted. Dashes and cross marks indicate sequence identities and insertion/deletion differences, respectively. These sequences are as follows: Platyfish *X. maculatus *(DQ866828), Tilapia *Oreochromis mossambicus *(AJ871147), Stickleback *Gasterosteus aculeatus *(group II contig 348), Tetraodon *Tetraodon nigroviridis *(AY332238), Medaka *Oryzias latipes *(scaffold 45), Zebrafish *Danio rerio *(NM_180970), Chicken *Gallus gallus *(NM_001031462), Human *Homo sapiens *(AF326275), and Mouse *Mus musculus *(NM_008559).

We determined the complete coding sequence and partial untranslated regions of the guppy *MC1R *gene. The complete coding sequence of the guppy *MC1R *gene contains 969 bp (+), which is predicted to encode a protein of 322 amino acids. The 969-bp guppy *MC1R *gene has the same length as the *MC1R *gene of a related species, the platyfish *Xiphophorus maculatus *(GenBank accession number DQ866828) [21]. To examine whether *MC1R *polymorphism is present in wild populations, we genotyped 270 individuals sampled from five wild populations in Trinidad. *MC1R *polymorphism was observed in two of these wild populations: Pitch Lake and Lower Guanapo River (Table 2). We identified two alleles of the guppy *MC1R *gene differing in length by 6 bp: one allele of 969 bp (+) (GenBank number AB563501); the other of 963 bp (Del) (GenBank number AB563502), which lacks two amino acids (Ser35 and Ser36) in the extracellular region. We determined the sequences using four indel-flanking primers. When we compared the sequences of homo- and heterozygous individuals, we found those from the heterozygotes to be unreadable. We were unable to detect any further alleles in any of the homozygotes examined in this study. Figure 3 shows an amino acid alignment of the extracellular region and the transmembrane region of *MC1R *that includes the deleted sites. We designated the homozygous genotype of the 963-bp (*Del*) length morph "*Del/Del*" and that of the 969-bp (+) length morph "*+/+*." In the Pitch Lake population, genotype frequencies were in approximate Hardy-Weinberg equilibrium (Table 2). A small non-significant deviation in the *+/Del *genotype was observed in the Lower Guanapo population (Ho = 0.040 and He = 0.113) (randomization test: p = 0.163).

**Table 2:** *MC1R *genotype frequencies of guppies in the wild

**Populations**	***MC1R *genotype**			**Allele frequencies**	
		
	***+/+***	***+/Del***	***Del/Del***	**P (+)**	**P (*Del*)**
	
Pitch Lake	68	20	3	0.85714	0.142857
Lower Aripo	45	0	0	1	0
Upper Aripo	36	0	0	1	0
Lower Guanapo	46	2	2	0.94	0.06
Upper Guanapo	48	0	0	1	0

The number of individuals shown is based on *MC1R *genotypes in each population.

Individuals with the 963-bp (*Del*) allele were mostly found in Pitch Lake. There was a significant difference in the frequency of the 963-bp (*Del*) allele among the five wild populations sampled (G = 48.243, df = 4, P < 0.0001). The frequencies of the 963-bp (*Del*) allele in Pitch Lake (PL) and the Lower Guanapo River (LG) were significantly higher than those in the other three populations (a simultaneous test procedure using the G-test: PL vs. LG, G = 4.822, df = 4, P = 0.306; PL and LG vs. Upper Aripo River, G = 20.1681, df = 4, P < 0.0004).

### Association between *MC1R *genotypes and black pigmentation

We examined the association between the genotypes of *MC1R *(*+/+*, *+/Del*, and *Del/Del*) and black pigmentation using the specimens obtained from aquarium shops. The typical black pigmentation of each *MC1R *genotype is shown in Figure 2. In males, *+/+ *individuals tended to exhibit a grayish body color and have black traits, whereas the *+/Del *and *Del/Del *individuals tended to exhibit a yellowish body color and have no black traits, which suggests that the yellowish pigmentation was highly visible in these individuals because black pigmentation was absent. In females, the *+/+ *individuals tended to exhibit a grayish body color, whereas the *Del/Del *individuals tended to exhibit a yellowish color. The *+/Del *females had phenotypes intermediate between those of *+/+ *and *Del/Del*. We found statistically significant differences in the frequencies of different genotypes (*+/+*:*Del/Del*, *+/+*:*+/Del*, and *+/+*:*+/Del*:*Del/Del*) between different body blackness (Grayish vs. Yellowish) and also in the frequencies of different genotypes (*+/+*:*Del/Del*, and *+/+*:*+/Del*:*Del/Del*) between different black traits (Black vs. None) (Table 3) (sequential Bonferroni corrections after Fisher's exact test: P < 0.05).

**Table 3:** Association between *MC1R *genotype and black pigmentation in guppies obtained from aquarium shops

(1) Body blackness				
*MC1R*	No. of males		No. of females	
		
Genotype	Grayish	Yellowish	Grayish	Yellowish

*+/*+	8	3	11	3
*+/Del*	3	14	4	7
*Del/Del*	1	7	1	5

(2) Black traits				

*MC1R*	No. of males		No. of females	
		
Genotype	Black	None	Black	None

*+/*+	8	3	8	5
*+/Del*	4	13	4	7
*Del/Del*	2	6	1	5

(1) For body blackness, "Grayish" guppies have black pigmentation and "Yellowish" guppies have no black pigmentation. (2) For black traits, "Black" and "None" indicate the presence and absence of black spots and lines, respectively.

### Association between *MC1R *genotypes and black pigmentation in F_1 _and F_2_

We obtained two F_2 _families (N = 39) from eight F_1 _individuals established by crossing two different homozygotes. The composition ratio of genotypes in the F_2 _individuals deviated from 1:2:1, with the *+/Del *genotype occurring at a frequency slightly greater than expected (overall ratio: 8:24:7). However, this bias was statistically non-significant (Fisher's exact test: P = 0.2)

All of the F_1 _individuals had grayish body color and black traits. The association between genotype and black pigmentation in the F_2 _individuals is shown in Table 4. There was no significant association between *MC1R *genotype and body blackness (Grayish vs. Yellowish) or between *MC1R *genotype and black trait (Black vs. None) (Fisher's exact test: P > 0.05) (Table 4).

**Table 4:** Association between *MC1R *genotype and black pigmentation in an F_2 _population

(1) Body blackness				
*MC1R*	No. of males		No. of females	
		
Genotype	Grayish	Yellowish	Grayish	Yellowish

*+/+*	3	3	2	0
*+/Del*	10	3	7	4
*Del/Del*	1	1	4	1

(2) Black traits				

*MC1R*	No. of males		No. of females	
		
Genotype	Black	None	Black	None

*+/+*	3	3	0	2
*+/Del*	11	2	4	7
*Del/Del*	2	0	0	5

(1) For body blackness, "Grayish" guppies have black pigmentation and "Yellowish" guppies have no black pigmentation. (2) For black traits, "Black" and "None" indicate the presence and absence of black spots and lines, respectively. (1, 2) All the F_2 _individuals from two families are included.

## Discussion

This study showed that there was *MC1R *polymorphism both within and among wild guppy populations. We identified two alleles of the *MCIR *gene that differed in length by 6 bp: 969 bp (+) and 963 bp (*Del*). The 969-bp (+) guppy *MC1R *gene is the same length as the *MC1R *gene of the platyfish, which is a related species. Thus, the 963-bp (*Del*) guppy *MC1R *allele may have lost a 6-bp sequence after divergence between the guppy and the platyfish. Although several studies have reported that polymorphism of color-related genes is maintained in wild fish populations [22-24], this is the first study to detect polymorphism in the color-related genes of guppies in the wild.

The present study showed that the frequency of the 963-bp (*Del*) allele of *MC1R *was higher in the Pitch Lake population than in the other sampled populations. Unlike guppies in the other populations, those in Pitch Lake are exposed to direct sunlight and high water temperatures, and the background color of the sediment in this habitat is black [25]. Similarly, a uniquely colored killifish (*Rivulus hartii*) with pink and white hue and two mottled darker patches has been reported in the Pitch Lake [26]. The relatively abundant lighter colored fish with *+/Del *and *Del/Del *genotypes may therefore be particularly conspicuous in the Pitch Lake environment. *MC1R *polymorphisms are associated with coloration in approximately half of the investigated species [9,27-35]. These authors mostly suggest that *MC1R *polymorphism contributes to the adaptation to divergent background colorations for crypsis. For example, in pocket mice (*Chaetodipus intermedius*), melanic *MC1R *mutants are found on dark lava fields, whereas light-colored mutants are found on light-colored rocks, suggesting that these variations in *MC1R *contribute to adaptive camouflage for predator avoidance on different backgrounds [28-30]. On the other hand, in several bird species, it has been reported that *MC1R *polymorphism is selected by sexual selection [31]. For example, in arctic skuas, females prefer dark males, and also those males having the same color as that of their parents, which contributes to positive assortative mating [32]. At this stage of our study, it is unclear whether the differences in allele frequencies across populations have been caused by random genetic drift and natural selection. To test the hypothesis that the differences are a result of natural or sexual selection, we ideally need to use statistical methods for detecting natural selection.

The present results suggest that there is a significant association between *MC1R *genotype and black pigmentation in the guppies obtained from aquarium shops (Table 3). These results indicate that the deletion of two amino acids in *MCIR *disturbed both overall grayish body blackness and black traits. *MC1R *plays crucial roles in controlling melanin synthesis [12]; therefore, by mutating *MC1R*, it is possible to increase or decrease melanin synthesis. Thus, deletion of two amino acids in the extracellular regions (Figure 3) might affect the affinity of *MC1R *for its ligands, i.e., *MSH *and β-defensin. If the affinity for these ligands is decreased, melanin synthesis is disturbed. In this study, most of the *Del/Del *individuals exhibited less distinct black pigmentation than *+/+ *individuals, and thus the deletion of two amino acids in *MC1R *might inhibit black pigment production. However, three of 14 *Del/Del *individuals had black pigmentation (Table 3), suggesting that other factors are involved in pigmentation. Furthermore, no association between *MC1R *genotype and black pigmentation could be found in the investigation using F_2 _individuals (Table 4). However, in this case, it is possible that, owing to an excess of heterozygous (+/*Del*) individuals, there was an insufficient number of homozygous F_2 _offspring to enable detection of an association.

There are two possible explanations to account for the lack of association between genotypes and phenotypes in the F_2 _population. First, the variously pigmented guppies found in aquarium fish shops are created by selective breeding. Thus, we cannot fully exclude the possibility that ancestral founders of the strains with white body color had a deletion allele of *MC1R *that did not affect black pigmentation. However, we used several different strains with white body color, and thus it is seems unlikely that the founders of these different strains simultaneously had a 963-bp (*Del*) allele of *MC1R*. Second, the existence of certain other modifier genes may have an effect on black pigmentation. Epistasis may alter the predicted association between genotype and phenotype. For example, in the pathway of melanin synthesis, *MC1R *activation and inhibition is influenced by melanocortin (e.g. *ACTH*, *MSH*, and β-defensin) and *ASIP *[9]. It is possible that these genes can modify the effects of the *MC1R *gene on melanin synthesis. In beach mice and flycatchers, pigmentation polymorphism in some populations can be explained by *MC1R *genotypes; however, in other populations, pigmentation polymorphism cannot be explained by the *MC1R *genotype, even though these populations exhibit similar pigmentation [33-35]. In addition, in this study, we were only able to obtain F_2 _individuals from two pairs (two crosses of *Del/Del *males and *+/+ *females), and thus there is a possibility that paternal or maternal genotypes affected the relationship between *MC1R *genotype and black pigmentation. In guppies, melanism may be controlled by multiple genes, which might be the genetic basis for the complex black pigmentation patterns in these fish. Further genetic studies are needed to confirm the relationships between *MC1R *genotypes and black pigmentation using established commercial strains and wild guppy populations.

In the present study, we identified two alleles of the *MCIR *gene that differ in length, namely, a 969-bp (+) and a 963-bp (*Del*) allele. *MC1R *polymorphism is present in wild populations, and we found that allele frequencies were significantly different among the different populations examined. Guppies with a 963-bp (*Del*) *MC1R *tend to exhibit less black pigmentation than those with a 969-bp (+) *MC1R*. Although the results of this study indicate the possibility of an association between *MC1R *genotype and black pigmentation, results from the study of F_2 _individuals did not allow us to confirm the link between *MC1R *genotype and black pigmentation. This suggests that there are other genes that modify the effects of the *MC1R *gene.

## Competing interests

The authors declare that they have no competing interests.

## Authors' contributions

AT carried out the molecular genetic studies and the laboratory experiments. HY and JY advised on the molecular genetic studies. CvO provided the samples of Trinidad population and advice on. MK supervised the research. AT and MK wrote the paper. All authors read and approved the final manuscript.

## References

[B1] Slominski A Tobin DJ Shibahara S Wortsman J Melanin pigmentation in mammalian skin and its hormonal regulation Physiological reviews 2004 84 4 1155 1228 10.1152/physrev.00044.2003 15383650

[B2] Hoekstra HE Genetics, development and evolution of adaptive pigmentation in vertebrates Heredity 2006 97 3 222 234 10.1038/sj.hdy.6800861 16823403

[B3] Houde AE Sex, Color and Mate Choice in Guppies 1997 Princeton University Press

[B4] Kotiaho JS Lebas NR Puurtinen M Tomkins JL On the resolution of the lek paradox Trends Ecol Evol 2008 23 1 1 3 10.1016/j.tree.2007.09.012 18077055

[B5] Olendorf R Rodd FH Punzalan D Houde AE Hurt C Reznick DN Hughes KA Frequency-dependent survival in natural guppy populations Nature 2006 441 7093 633 636 10.1038/nature04646 16738659

[B6] Hughes KA Du L Rodd FH Reznick DN Familiarity leads to female mate preference for novel males in the guppy, Poecilia reticulata Anim Behav 1999 58 4 907 916 10.1006/anbe.1999.1225 10512664

[B7] Blows MW Brooks R Kraft PG Exploring complex fitness surfaces: multiple ornamentation and polymorphism in male guppies Evolution Int J Org Evolution 2003 57 7 1622 1630 10.1111/j.0014-3820.2003.tb00369.x 12940366

[B8] Crispo E Bentzen P Reznick DN Kinnison MT Hendry AP The relative influence of natural selection and geography on gene flow in guppies Mol Ecol 2006 15 1 49 62 10.1111/j.1365-294X.2005.02764.x 16367829

[B9] Ducrest AL Keller L Roulin A Pleiotropy in the melanocortin system, coloration and behavioural syndromes Trends Ecol Evol 2008 23 9 502 510 10.1016/j.tree.2008.06.001 18644658

[B10] Endler JA Houde AE Geographic variation in female preferences for male traits in poecilia reticulata Evolution 1995 49 3 14 10.2307/2410270 28565093

[B11] Winemiller KO Leslie M Roche R Phenotypic variation in male guppies from natural inland populations: an additional test of Haskins' sexual selection/predation hypothesis Environmental Biology of Fishes 1990 29 179 191 10.1007/BF00002218

[B12] Jackson IJ Homologous pigmentation mutations in human, mouse and other model organisms Hum Mol Genet 1997 6 10 1613 1624 10.1093/hmg/6.10.1613 9300652

[B13] Lin JY Fisher DE Melanocyte biology and skin pigmentation Nature 2007 445 7130 843 850 10.1038/nature05660 17314970

[B14] Pointer MA Mundy NI Testing whether macroevolution follows microevolution: are colour differences among swans (Cygnus) attributable to variation at the MCIR locus? BMC Evol Biol 2008 8 249 10.1186/1471-2148-8-249 18789136 PMC2553801

[B15] Ito S Wakamatsu K Quantitative analysis of eumelanin and pheomelanin in humans, mice, and other animals: a comparative review Pigment Cell Res 2003 16 5 523 531 10.1034/j.1600-0749.2003.00072.x 12950732

[B16] Thompson JD Gibson TJ Plewniak F Jeanmougin F Higgins DG The CLUSTAL_X windows interface: flexible strategies for multiple sequence alignment aided by quality analysis tools Nucleic Acids Research 1997 25 24 4876 4882 10.1093/nar/25.24.4876 9396791 PMC147148

[B17] Doyle JJ Doyle JL A rapid DNA isolation procedure for small quantities of fresh leaf tissue Phytochemical Bulletin 1987 19 11 15

[B18] Kodric-Brown A Sexual dichromatism and temporary color changes in the reproduction of fishes American Zoologist 1998 38 1 70 81

[B19] Sokal R Rohlf F Biometry: The principles and practice of statistics in biological research 1995 3 New York: W. H. Freeman and Company

[B20] Team RDC R: A language and environment for statistical computing 2008 Vienna, Austria: R Foundation for Statistical Computing

[B21] Selz Y Braasch I Hoffmann C Schmidt C Schultheis C Schartl M Volff JN Evolution of melanocortin receptors in teleost fish: the melanocortin type 1 receptor Gene 2007 401 1-2 114 122 10.1016/j.gene.2007.07.005 17707598

[B22] Gross JB Borowsky R Tabin CJ A novel role for Mc1r in the parallel evolution of depigmentation in independent populations of the cavefish Astyanax mexicanus PLoS genetics 2009 5 1 e1000326 10.1371/journal.pgen.1000326 19119422 PMC2603666

[B23] Roberts RB Ser JR Kocher TD Sexual Conflict Resolved by Invasion of a Novel Sex Determiner in Lake Malawi Cichlid Fishes Science 2009 19797625 10.1126/science.1174705 PMC3174268

[B24] Miller CT Beleza S Pollen AA Schluter D Kittles RA Shriver MD Kingsley DM cis-Regulatory changes in Kit ligand expression and parallel evolution of pigmentation in sticklebacks and humans Cell 2007 131 6 1179 1189 10.1016/j.cell.2007.10.055 18083106 PMC2900316

[B25] Kenny JS Views from the bridge: A memoir on the freshwater fishes of Trinidad 1995 Port of Spain: The University of the West Indies Press

[B26] Mohammed RS McMullan MJ Schelkle B van Oosterhout C Colour Variation of an Individual of Hart's Rivulus (Rivulus hartii) found in a Habitat Rich in Polycyclic Aromatic Hydrocarbons in the Pitch Lake of Trinidad Ecologia Balkanica 2010 2 61 63

[B27] Rosenblum EB Hoekstra HE Nachman MW Adaptive reptile color variation and the evolution of the Mc1r gene Evolution Int J Org Evolution 2004 58 8 1794 1808 10.1111/j.0014-3820.2004.tb00462.x 15446431

[B28] Hoekstra HE Nachman MW Different genes underlie adaptive melanism in different populations of rock pocket mice Mol Ecol 2003 12 5 1185 1194 10.1046/j.1365-294X.2003.01788.x 12694282

[B29] Nachman MW Hoekstra HE D'Agostino SL The genetic basis of adaptive melanism in pocket mice Proceedings of the National Academy of Sciences of the United States of America 2003 100 9 5268 5273 10.1073/pnas.0431157100 12704245 PMC154334

[B30] Hoekstra HE Drumm KE Nachman MW Ecological genetics of adaptive color polymorphism in pocket mice: geographic variation in selected and neutral genes Evolution Int J Org Evolution 2004 58 6 1329 1341 10.1111/j.0014-3820.2004.tb01711.x 15266981

[B31] Mundy NI A window on the genetics of evolution: MC1R and plumage colouration in birds Proc Biol Sci 2005 272 1573 1633 1640 10.1098/rspb.2005.3107 16087416 PMC1559852

[B32] Mundy NI Badcock NS Hart T Scribner K Janssen K Nadeau NJ Conserved genetic basis of a quantitative plumage trait involved in mate choice Science 2004 303 5665 1870 1873 10.1126/science.1093834 15031505

[B33] Hoekstra HE Hirschmann RJ Bundey RA Insel PA Crossland JP A single amino acid mutation contributes to adaptive beach mouse color pattern Science 2006 313 5783 101 104 10.1126/science.1126121 16825572

[B34] Steiner CC Rompler H Boettger LM Schoneberg T Hoekstra HE The genetic basis of phenotypic convergence in beach mice: similar pigment patterns but different genes Mol Biol Evol 2009 26 1 35 45 10.1093/molbev/msn218 18832078

[B35] Uy JA Moyle RG Filardi CE Cheviron ZA Difference in plumage color used in species recognition between incipient species is linked to a single amino acid substitution in the melanocortin-1 receptor Am Nat 2009 174 2 244 254 10.1086/600084 19489704

